# A novel alpha-1 antitrypsin gene variant in a patient with Kartagener's syndrome: a case report

**DOI:** 10.3325/cmj.2024.65.450

**Published:** 2024-10

**Authors:** Levent Ozdemir, Burcu Ozdemir, Savaş Gegi̇n

**Affiliations:** Department of Pulmonology, Samsun Training and Research Hospital, Samsun, Turkey

## Abstract

Alpha-1 antitrypsin deficiency (AATD) is a rare autosomal co-dominant disease caused by mutations in the SE*RPINA1* gene. The alleles most frequently associated with AATD are protease inhibitors S and Z. Here, we report on a 35-year-old woman diagnosed with Kartagener's syndrome and subsequently referred for bronchiectasis testing. She was identified with a hitherto unreported AATD mutation: a heterozygous variant rs1460874866 in a previously undefined exon 4 (NM_001127701.1) of the SE*RPINA1* gene. Although Kartagener's syndrome is a genetic cause of bronchiectasis, patients with this syndrome are recommended to undergo AATD testing.

Kartagener's syndrome is a genetic condition characterized by the triad of bronchiectasis, *situs inversus*, and sinusitis. It is inherited as an autosomal recessive trait (1). Bronchiectasis is one of the pulmonary symptoms associated with a deficiency in alpha-1 antitrypsin (AAT), a primary protease inhibitor that protects the lung parenchyma from proteolytic assaults (2). AAT deficiency (AATD) is a prevalent but poorly understood autosomal co-dominant disease characterized by low serum AAT levels caused by mutations in the SE*RPINA1* gene. Despite the identification of over 150 SE*RPINA1* variants to date, novel genetic variants have been frequently discovered. In the past decade, researchers have identified 22 new genetic variants associated with SE*RPINA1* (3). The 14 allele variants most frequently associated with AAT deficiency are listed in Table 1.

**Table 1:** Frequently detected allelic variants associated in SE*RPINA1.*

Frequently detected allelic variants	Nucleotide change	Amino acid change HGVS nomenclature	Amino acid change (alternative name) in the mature protein or affected exons in large indels	SNP code
PI*I	c.187C>T	p.(Arg63Cys)	Arg39Cys	rs28931570
PI*M procida	c.194T>C	p.(Leu65Pro)	Leu41Pro	rs28931569
PI*M malton	c.227_229delTCT	p.(Phe76del)	Phe52del	rs775982338
PI*S iiyama	c.230C>T	p.(Ser77Phe)	Ser53Phe	rs55819880
PI*Q0 granite falls	c.552delC	p.(Tyr184*)	Tyr160*	rs267606950
PI*Q0 west	c.646 + 1G>T	NA	NA	rs751235320
PI*Q0 bellingham	c.721A>T	p.(Lys241*)	Lys217*	rs199422211
PI*F	c.739C>T	p.(Arg247Cys)	Arg223Cys	rs28929470
PI*lowell	c.839A>T	p.(Asp280Val)	Asp256Val	rs121912714
PI*S	c.863A>T	p.(Glu288Val)	Glu264Val	rs17580
PI*Z	c.1096G>A	p.(Glu366Lys)	Glu342Lys	rs28929474
PI*Q0 mattawa	c.1130dupT	p.(Leu377Phefs*24)	Leu353Phefs*24	rs763023697
PI*Q0 clayton	c.1158dupC	p.(Glu387Argfs*14)	Glu363Argfs*14	rs764325655
PI*M heerlen	c.1178C>T	p.(Pro393Leu)	Pro369Leu	rs199422209

*Abbreviations: HGVS – Human Genome Variation Society; SNP – single-nucleotide polymorphism.

Here, we report on a patient with Kartagener's syndrome who was diagnosed with AAT genotype deficiency and had a previously unidentified heterozygous variant rs1460874866 in the exon 4 (NM_001127701.1) of the SE*RPINA1* gene.

## CASE REPORT

**Figure 1:**
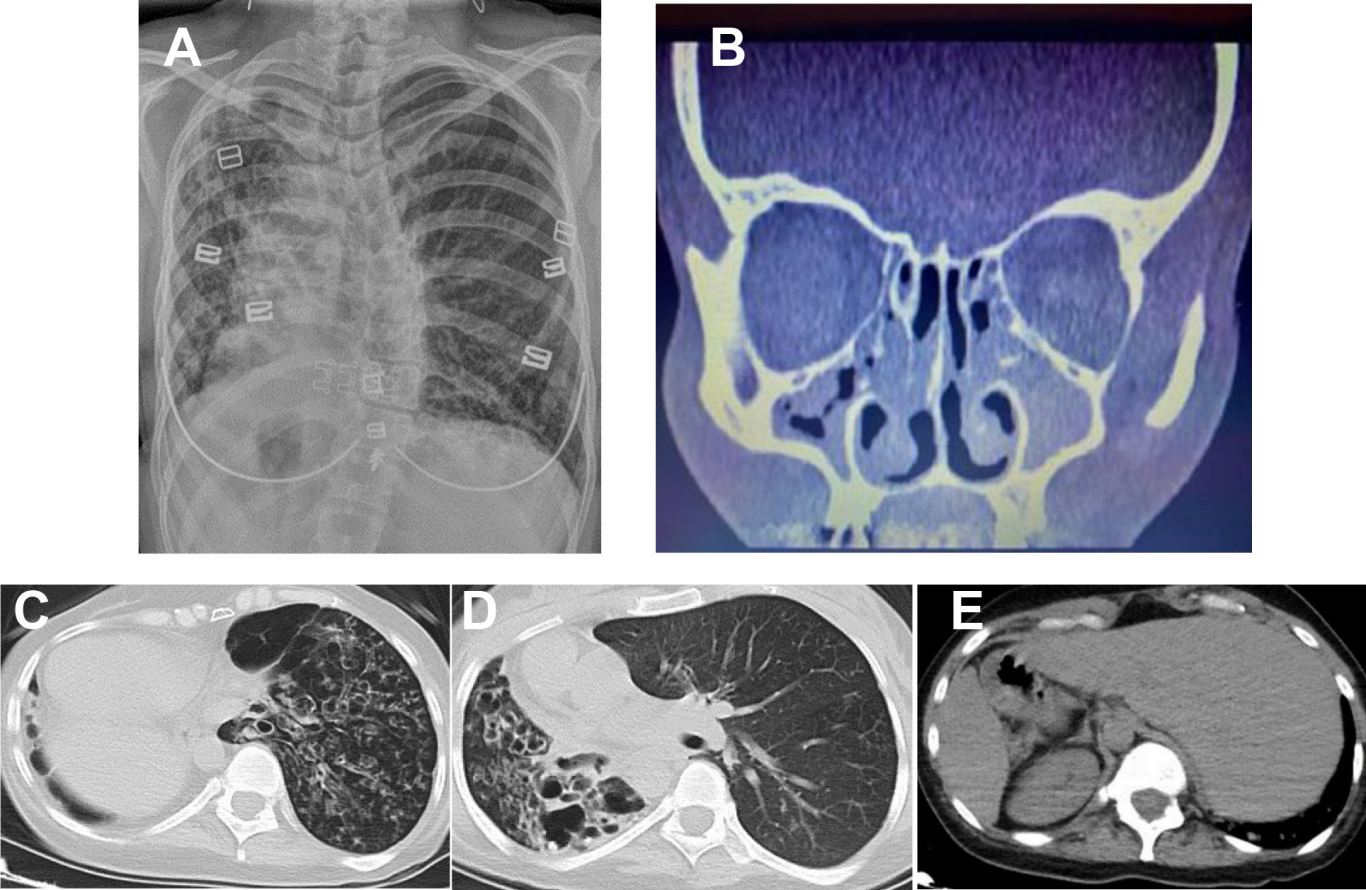
(**A**) Chest x-ray: heart and fundus air shadow on the right; (**B**) paranasal sinus tomography indicating sinusitis; (**C-D**) thorax tomography indicating cystic bronchiectasis in both lungs; (**E**) upper abdomen tomography: the spleen and stomach on the right, the liver on the left.

A 35-year-old woman was examined at our Chest Diseases Polyclinic in February 2023 due to coughing, wheezing, sputum production, and shortness of breath. She had been diagnosed with Kartagener's syndrome in her childhood and had undergone two operations for bronchiectasis. She had no history of smoking, and her family history was unremarkable. The respiratory system examination revealed prominent bilateral rales in the right lower zone, with rhonchi present in all zones. Her cardiac apex pulse was observed on the right. The chest x-ray showed the fundal air shadow of the heart and stomach on the right side. Bronchiectatic areas were identified in the right middle-lower and left lower zone. Paranasal sinus tomography revealed sinusitis. On thorax tomography, cystic bronchiectasis was visible in the left lower lobe and the right lung, along with dextrocardia and volume loss. Upper abdominal tomography showed the liver to be located on the left, while the spleen and stomach were on the right (Figure 1A-E). The laboratory parameters were within the reference range. The results of the pulmonary function test were as follows: forced vital capacity 0.84 (26%) and forced expiratory volume in one second 0.81 (30%). Alpha-1 antitrypsin level was 1.1 g/L (reference range: 0.9-2 g/L).

**Figure 2:**
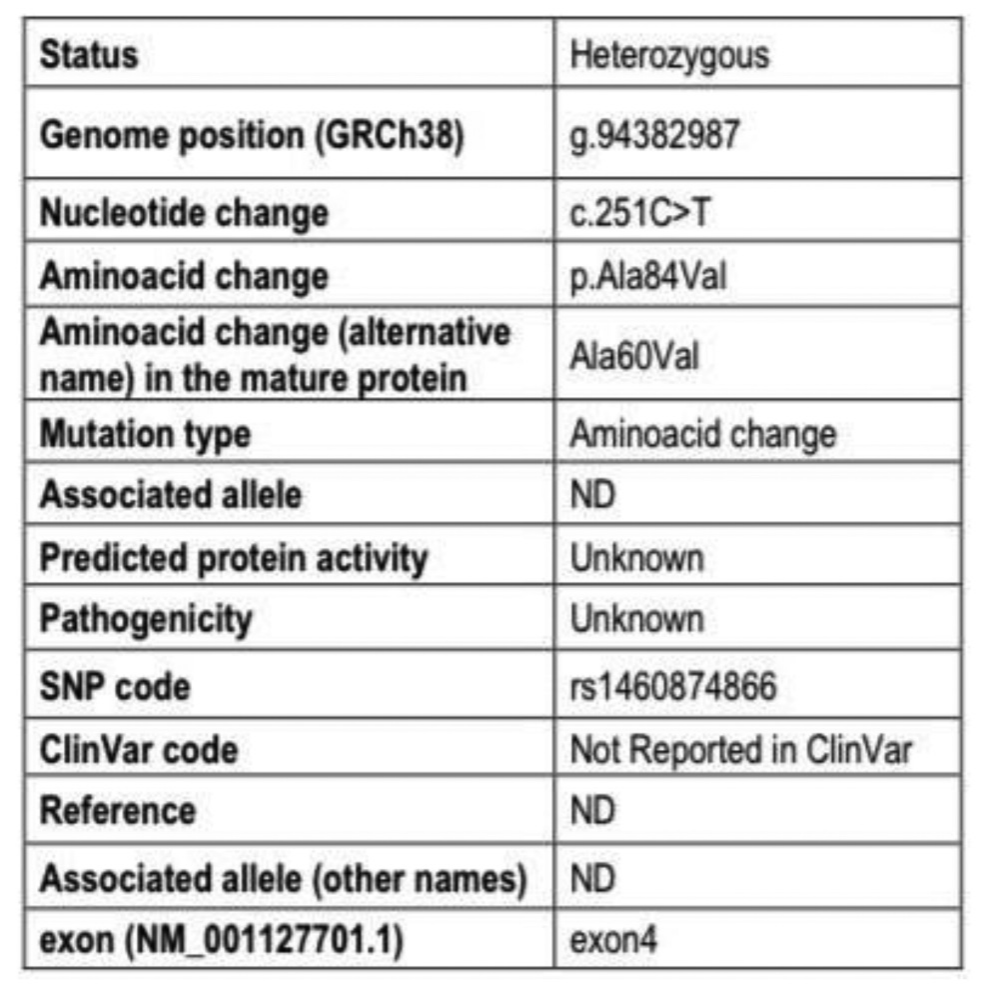
The sequencing results obtained for the novel gene variant observed in our patient with Kartagener's syndrome.

**Figure 3:**
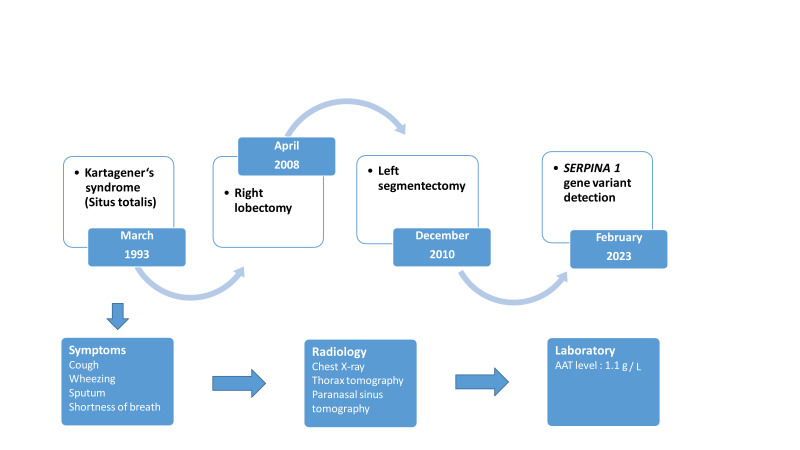
The timeline of diagnostic tests and events. AAT – alpha-1 antitrypsin.

As it is recommended that all patients with Kartagener’s syndrome are tested for AATD, the patient was referred for SE*RPINA1* genotyping. Fingerprick blood was collected. The genomic DNA from desiccated drop blood samples was replicated by polymerase chain reaction and hybridized with allele-specific probes by using the Luminex xMAP technology (Luminex, Austin, TX, USA). The screening identified a heterozygous variant rs1460874866 in a previously undefined exon 4 (NM_001127701.1) (Figure 2). The patient continues to be followed up regularly at our clinic. The timeline of events and diagnostic procedures is illustrated in Figure 3.

## DISCUSSION

Bronchiectasis is a chronic respiratory disease characterized by irreversible pathological dilation of the bronchia. Symptoms include recurrent respiratory tract infections, sputum, and cough. The components of the bronchiectasis etiology include primary ciliary dyskinesia (Kartagener's syndrome) (4), congenital AATD, and cystic fibrosis (5). Bronchiectasis is one of the pulmonary symptoms associated with AATD (6). The prevalence of AATD is 20/100 000 (7). Regardless of smoking status, the World Health Organization recommends that all patients with chronic obstructive pulmonary disease and emphysema undergo testing for AATD. Additionally, individuals who have siblings with liver disease, bronchiectasis, partially reversible asthma, necrotizing panniculitis, and the proteinase inhibitor ZZ (PiZZ) allele are advised to undergo the test (8).

Limited research has been conducted to assess the prevalence of AATD in patients with bronchiectasis. The PiZZ phenotype was detected in 0.5% of 1600 patients, the proteinase inhibitor SZ phenotype in 0.4%, and the proteinase inhibitor MZ phenotype in 3%. Severe AATD was identified in less than 1% of patients with bronchiectasis (9). Although the identification of rare variants is on the rise, particularly in patients with low AAT concentrations, the clinical significance of these variants remains unknown (3). In our case, the treatment indication was not provided for the AAT level, as it was within the reference range. Our investigation obtained no evidence regarding the impact of the detected genetic variant on the patient's health.

In conclusion, we reported on a patient with Kartagener's syndrome identified with an AATD variant that has not been previously identified in our literature review or the reference laboratory archive. Although Kartagener's syndrome is a genetic cause of bronchiectasis, it is recommended that these patients undergo AATD testing.
